# An Efficient Sonochemical Synthesis of Novel Schiff's Bases, Thiazolidine, and Pyrazolidine Incorporating 1,8-Naphthyridine Moiety and Their Cytotoxic Activity against HePG2 Cell Lines

**DOI:** 10.1155/2014/587059

**Published:** 2014-02-25

**Authors:** N. S. Ahmed, K. O. Alfooty, S. S. Khalifah

**Affiliations:** ^1^Chemistry Department, Faculty of Science, King Abdulaziz University, P.O. Box 80203, Jeddah 21589, Saudi Arabia; ^2^Medicinal Chemistry Department, National Research Center, Dokki, Cairo 12622, Egypt

## Abstract

Novel Schiff's bases **4a–e**, **5a**, **5b**, and **6**, thiazolidine **7a–d**, and pyrazolidine **8** have been synthesized using the versatile synthon 4-hydroxy-2,7-dimethyl-1,8-naphthyridine **1**. Reactions carried out under ultrasound irradiation showed higher rates and yields than those done under silent conditions. The newly synthesized compounds were evaluated for HepG2 cell growth inhibition. The results obtained revealed that the tested compounds possess inhibitory effect on the growth of HepG2 liver cancer cells. The results were compared to doxorubicin as a reference drug (IC_50_: 0.04). Compounds **4a** and **7b** showed the highest inhibition activity against the HepG2 cell line (IC_50_: 0.047 and 0.041 µM, resp.) among all the tested compounds.

## 1. Introduction

**Figure 1:**
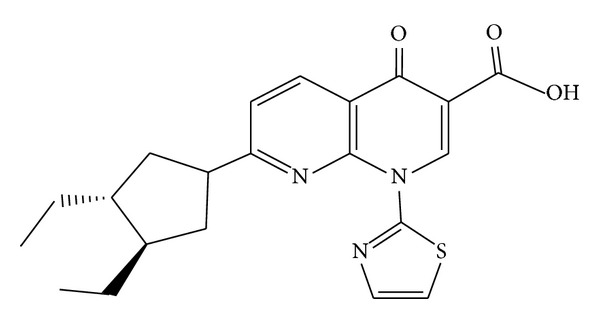
Vosaroxin.

Substituted nitrogen heterocycles are common motifs in biological and pharmaceutical science [1]. For example, 1,8-naphthyridine derivatives have promising medicinal properties, including anti-HIV [2], anticancer [3], anti-inflammatory [4], antimalarial [5], antibacterial [6], antiprotozoal [7], antimycobacterial [8], and antiplatelet [9] activity. In addition 1,8-naphthyridine derivatives were found to display cytotoxic activity against the murine P388 leukemia cell line when changes were carried out at the N-1 and C-7 positions [10, 11]. Moreover, it was recently found that the 1,8-naphthyridine derivative vosaroxin (formerly SNS-595, AG-7352, AT-3639, or voreloxin) was found to have potential anticancer activity. This drug (Figure 1) is believed to exert its action via topoisomerase II inhibition [12]. Topoisomerase II is one of the well-known targets for antitumor agents like doxorubicin, etoposide, ellipticine, and amsacrine [13]. We have reported in a previous article that 1,8-naphthyridine substituted with Mannich bases, *N*-*β*-glycosides, and Schiff's bases showed potent cytotoxic activity against the HepG2 cell line [14].

The application of ultrasound in synthetic organic chemistry became crucial. Sonochemistry is a new trend in organic chemistry, offering a versatile pathway for a wide variety of syntheses. A large number of organic reactions can be carried out under ultrasonic irradiation in high yields, short time, and mild conditions [15–20]. We are motivated by the aforementioned findings, our ongoing endeavors in the development of convenient synthetic approaches for the construction of biologically active heterocycles, and the growing interest in sonochemistry [21]. Our strategy is to develop a facile sonochemical synthesis and high yield procedure to prepare some novel 1,8-naphthyridine-4-oxyacetohydrazide Schiff's bases and 1,8-naphthyridine-4-oxyacetamide incorporated into thiazolidine and pyrazolidine moieties and the investigation of their biological activities in suppressing the growth of HepG2 liver cancer cells.

## 2. Results and Discussion

### 2.1. Chemistry

**Scheme 1:**
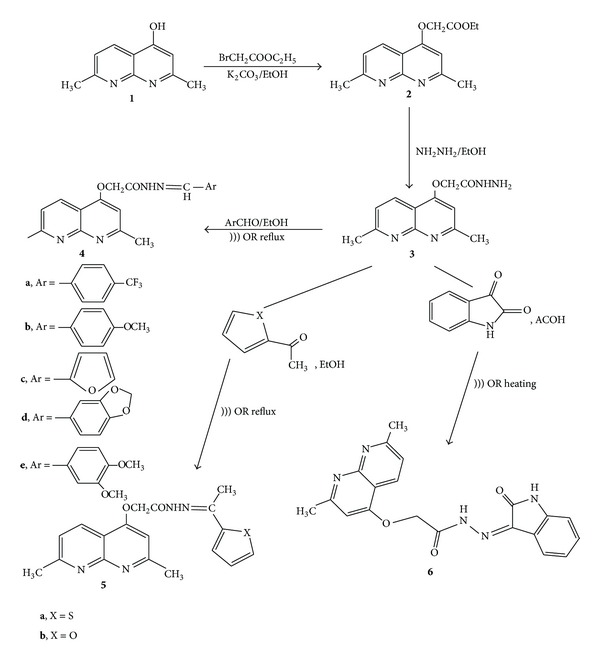


The starting material, namely, 2-(2,7-dimethyl-1,8-naphthyridin-4-yloxy) acetohydrazide **3**, was synthesized by the reaction of 4-hydroxy-2,7-dimethyl-1,8-naphthyridine **1** [22] with ethyl bromoacetate in the presence of anhydrous potassium carbonate [23] in absolute ethanol under reflux to give the nonisolated ester **2**. The ester **2** reacted directly with hydrazine hydrate in refluxing ethanol which afforded the acid hydrazide **3** (Scheme 1). The structure of the acid hydrazide was established on elemental analysis and from spectral data. The IR spectra revealed the two absorption bands at 3338 and 3193 cm^−1^ which correspond to -NHNH_2_ and a band at 1671 cm^−1^ due to amidic carbonyl group. Its ^1^H NMR spectrum showed two D_2_O exchangeable signals due to NH_2_ and NH protons at *δ* 3.99 and 9.28, respectively. Two singlets due to 2 methyl groups at *δ* 2.44 and 2.61 were observed. A broad singlet is present at *δ* 5.20 for methylene protons in addition to the 3 aromatic protons of naphthyridine at *δ* 6.18, 7.20, and 8.46. The hydrazide obtained **3 **was then condensed with different aromatic aldehydes, ketones, and isatine, in absolute ethanol under ultrasound irradiation at 60–65°C, and produced the corresponding Schiff's bases **4a–e**, **5a**, **5b**, and **6**, respectively (Scheme 1).

**Figure 2:**
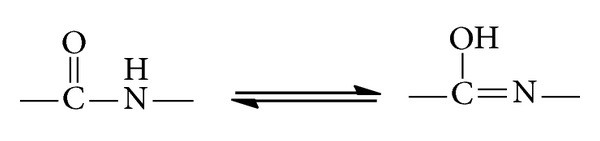


**Scheme 2:**
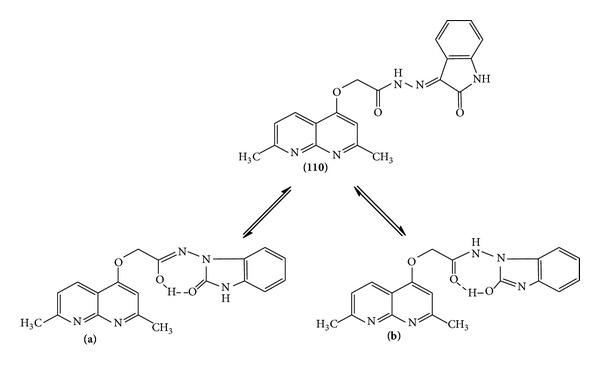


In the ^1^H NMR spectra of Schiff's bases **4a–e**, the disappearance of the broad singlet band at *δ* 3.99 which corresponds to NH_2_ protons and an additional set of signals assigned to the –N=CH– group in the range *δ* 7.95–8.12 were observed. This observation confirmed the condensation between the amino group of the hydrazide and the carbonyl compounds. The structure of Schiff's bases derived from aromatic ketones **5a**, **b **was established on the basis of its elemental analysis and spectral data. The ^1^H NMR spectrum of **5a** revealed a new singlet signal for an extra CH_3_ group at *δ* 2.17 beside the 3 protons of the thiophene ring at *δ* 7.06, 7.32, and 7.37. Moreover, an interesting observation appeared in the IR spectra of Schiff's base derived from the isatin **6**, where broad absorption bands shown at 3214–3454 cm^−1^ were attributed to the contribution of enolic OH and NH groups. This observation is consistent with similar reported compounds containing the isatin moiety [24]. The enolic character in this compound was further confirmed by the ^1^H NMR at 600 MHz. Four signals centered in the range of *δ* 10.8 to 12.27 were assigned to amidic iminol structures [25] (Scheme 2 and Figure 2).

To find the specific effect of ultrasound on this reaction, all previously mentioned reactions were carried out under the same conditions in the absence of ultrasound irradiation (Table 1). The data cited in Table 1 show that the reaction time increased while the product yields slightly decreased in the absence of ultrasonic irradiation. These results confirm that the ultrasonic irradiation played a crucial role in the enhancement of the rapid synthesis of Schiff's bases. Based on the above findings, we further extended our study to investigate the reactivity of compounds **4a–e** which are considered as suitable precursors for the synthesis of novel [4-((oxyacetamido)thiazolidin-3-yl)2,7-dimethyl-1,8-naphthyridine] derivatives **7a–d** and pyrazolidine derivative **8**.

**Scheme 3:**
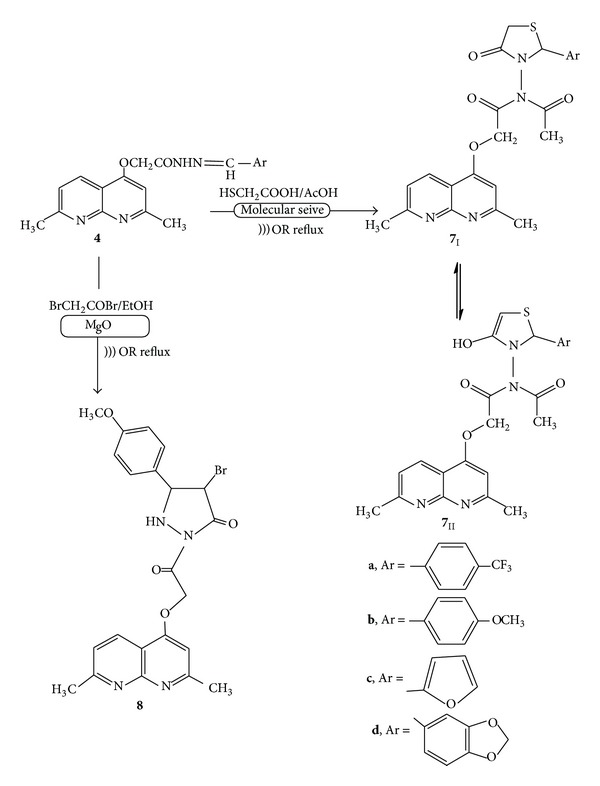


Treatment of **4a–d** with thioglycolic acid in acetic acid under “silent” conditions resulted in cyclocondensation giving the corresponding thiazolidinone derivatives **7a–d**. Upon repeating the reaction using ultrasonic irradiation instead of the classical method, the formation of the desired product in a shorter time (as examined by TLC) without an improvement in yield was observed. However, a catalyzed ultrasound irradiation process using molecular sieve (4 Å) resulted in a good yield from **7a–d** in an even shorter time. The structures of the compounds** 7a–d** were established on the basis of their elemental analysis, IR, ^1^H NMR, ^13^C NMR, and mass spectral data. Compounds **7a–d **may be formulated as the *oxo*-form **7**_**I**_ **a–d** or its tautomeric *enol*-form **7**_**II**_ **a–d** (Scheme 3).

IR spectra of the isolated products revealed the predominance of the *enol*-form **7**_**II**_ **a–d** due to the existence of strong absorption peaks in the region of *ν* = 3360–3380 cm^−1^ which corresponds to cyclic *enol*, while the amidic carbonyl absorption appeared at *ν* = 1680 cm^−1^. The ^1^H NMR of compound **7a** showed a new singlet signal at *δ* 2.07 due to an acetyl group, two doublet signals at *δ* 3.71 and 3.90 for CH_2_– in thiazolidine, and CH– thiazolidine appeared at *δ* 8.29. The time of the reaction and the product yields are cited in Table 2, which also shows that the catalyzed ultrasound technique reduced the time of the reactions from several hours to minutes and improved the product yields from 40–49% (under conventional conditions) to 89–93%. Treatment of Schiff's base **4b** with bromoacetyl bromide in ethanol and the presence of MgO as a solid base catalyst under ultrasonic irradiation afforded only one isolable product (as examined by TLC) identified as pyrazolidin-3-one core structure **8 **in 96% yield within 10 minutes. The same reaction was carried out in the absence of ultrasonic irradiation and gave just 45% yield, in a much longer reaction time (72 h). The structure of **8 **was established on the basis of its elemental analysis and spectral data. For example, its mass spectrum revealed a molecular ion peak at *m*/*z* 485 and at 487 for M^+^+2 and; its ^1^H NMR revealed two doublet signals due to the pyrazolidine 2–CH at *δ* 2.99 and 3.32.

In general, the improvement induced by ultrasound in the abovementioned reaction is based on the well-established cavitation theory [26]. The formation of Schiff's bases follows a false sonochemistry type according to the sonochemical reactions classification of Luche [27, 28]. The cavitation effect provides the mechanical energy for all subsequent chemical reactions, including bond scission induced by viscous frictional forces. In the present study, the substantial improvement induced by ultrasound irradiation in the reactions involving the formation of thiazolidine and pyrazolidine was assisted by the presence of solid catalysts. The pronounced enhancement of the ultrasound effect in the presence of solid catalysts is mainly due to cavitation in the liquid-solid system [29, 30]. The cavitation occurred in the liquid near the solid surface of the catalyst, resulting in a cavity collapse that generates high-speed jets of liquid, which hit the surface of the catalyst with tremendous force. This process could generate more reaction active sites at the catalyst's surface, which led to a pronounced increase in the reaction rate and the production of a high percentage yield in short reaction times.

### 2.2. Pharmacology

Preliminary screening of some selected compounds is given in Table 3. It is clear from the data cited in Table 3 that the tested compounds exhibit a moderate to strong growth inhibition activity on the tested cell line between 0.041 and 0.094 *μ*M concentrations in comparison to the known anticancer drug doxorubicin (DOX.). The cytotoxic activity of the selected derivatives on liver HepG2 cell lines, in comparison to the traditional anticancer drug DOX, revealed that compounds **4a** and **7b** were the most active and induced a marked growth inhibition against HepG2 when compared to DOX (**4a** and **7b** IC_50_ equal 0.047 and 0.041 *μ*M, resp., whereas DOX was 0.04 *μ*M).

## 3. Conclusion 

A class of novel Schiff's bases, thiazolidine and pyrazolidine, incorporated into 1,8-naphthyridine nucleus under both sonication and classical conditions were synthesized successfully. Ultrasonic irradiation resulted in pronounced improvements in both rates and yield of reactions. The use of solid catalysts enhances the efficacy of sonication and leads to the formation of high percentage yields in shorter reaction times. The cytotoxicity screening of some selected new compounds revealed that the selected compounds showed reasonable antitumor activity against the HepG2 cancer cell line in comparison to the traditional anticancer drug DOX. Among all the compounds tested, **4a** and **7b** were found to have the highest inhibitory activity against the HepG2 cell line with IC_50_ values of 0.047 and 0.041 *μ*M, respectively.

## 4. Experimental

### 4.1. Chemistry

#### 4.1.1. General

All melting points were measured on a Mel-Temp apparatus and were uncorrected. Thin layer chromatography (TLC) was performed on aluminum silica gel 60 F_254_ (E-Merk). The spots were detected by iodine and UV light absorption. IR spectra were recorded on a FTIR, Perkin Elmer SP 100 spectrometer. ^1^H NMR and ^13^C NMR spectra were recorded on Burker WM 350 and 600 MHz spectrometers using TMS (0.00 ppm) or the signal of the deuterated solvent was used as an internal standard. Chemical shift (*δ*) is given in ppm relative to the signal for TMS as standard and the coupling constant in Hz. Mass spectra were recorded on a Shimadzu GCMS-QP 1000 EX mass spectrometer at 70 e.v. Sonication was performed by Daihan (Wiseclean, D-40 kHz). Microanalysis was performed using a Perkin Elmer elemental analyzer at the Faculty of Science, King Abdul Aziz University. Biological activity tests were performed at the National Cancer Institute, Cairo, Egypt.

#### 4.1.2. Typical Procedure for the Reactions

*4.1.2.1. Synthesis of Acid Hydrazide Derivative 3. *A mixture of **1 **[22] (5 gm, 0.03 moL), ethyl bromoacetate (3.33 mL, 0.03 moL), and (5 gm) anhydrous potassium carbonate in abs. ethanol (13 mL) was refluxed for 3 h. The reaction mixture was filtered hot and the solvent was evaporated under vacuum. The residue obtained was sufficiently pure for the next step. Hydrazine hydrate (12 mL, 99%) was added to the forgoing residue and 20 mL abs. ethanol; and the reaction mixture was heated under reflux for 3 h. The reaction mixture was cooled at room temperature (r.t.) and the precipitate formed was filtered off, dried, and crystallized from ethanol to give the corresponding 2-(2,7-dimethyl-1,8-naphthyridin-4-yloxy) acetohydrazide as pale yellow crystals (55% yield); m.p. 275-276°C. FTIR: 3338, 3193 (–NHNH_2_), 1671 (C=O amidic), 1602 cm^−1^ (C=N); ^1^H NMR (600 MHz, DMSO-*d*_6_) *δ*_H_: 2.44, 2.61 (6H, 2s, 2CH_3_), 3.99 (2H, br.s, NH_2_, D_2_O exchangeable), 5.20 (2H, s, –OCH_2_), 6.18 (1H, S, C_3_–H), 7.20 (1H, *d*, C_6_–H, *J *= 7.8 Hz), 8.46 (1H, *d*, C_5_–H, *J* = 7.8 Hz), 9.28 (1H, br.s, –CONH D_2_O exchangeable); ^13^C NMR (EtOD) *δ*_C_: 24.01, 25.09, 46.63, 112.16, 118.17, 121.02, 136.19, 151.09, 155.19, 163.50, 168.83, 179.16; MS (*m*/*z*): 246 M^+*∙*^ (found: C, 58.53; H, 5.93; N, 22.55. C_12_H_14_N_4_O_2_ requires C, 58.13; H, 5.73; N, 22.75).

*4.1.2.2 Synthesis of 2-(2,7-Dimethyl-1,8-naphthyridin-4-yloxy) Acetohydrazide—Aromatic Aldehydes Schiff's Bases (* ***7a–e***)

*4.1.2.2.1. Method A: Silent Reactions.* An equimolar mixture of **3** (0.5 gm, 0.002 moL) and the appropriate aromatic aldehyde (0.002 moL) in 10 mL absolute ethanol was stirred under reflux for a suitable time (until the disappearance of starting materials as examined by TLC). The reaction mixture was concentrated and cooled and the crude product, so-formed, was collected by filtration and recrystallized from ethanol to give the title compound **4a–e**. 

*4.1.2.2.2. Method B: Sonicated Reactions.* To a solution of 2-(2,7-dimethyl-1,8-naphthyridin-4-yloxy) acetohydrazide **3 **(0.002 moL) in ethanol (10 mL) and appropriate aromatic aldehyde (0.002 moL) in a 100 mL Erlenmeyer flask. The mixture was subjected to ultrasound irradiation at 60–65°C for suitable time (cf. Table 1) until the starting material was no longer detectable by TLC. The precipitate formed was filtered off and recrystallized from ethanol to produce the corresponding Schiff's bases **4a–e**.

The synthesized compounds (**4a–e**) with their physical data are listed below.

*2-(2,7-Dimethyl-1,8-naphthyridin-4-yloxy)-N*′*-(4-(trifluoromethyl)benzylidene)-acetohydrazide (* ***4a*** *). *Off-white crystals; m.p. 282–284°C. FTIR: 3200 (–NH), 1679 (C=O amidic), 1640 (C=N), 1605 (C=C); ^1^H NMR (600 MHz, CDCl_3_: DMSO-*d*_6_) *δ*_H_: 2.16, 2.48 (6H, 2s, 2CH_3_), 5.78 (2H, br.s, –CH_2_), 6.22 (1H, s, C_3_–H), 7.20 (1H, *d*, C_6_–H, *J* = 7.8 Hz), 7.68, 7.88 (4H, 2*d*, *p*-disubstituted benzene, *J* = 8.4 Hz), 8.12 (1H, s, –N=CH), 8.49 (1H, *d*, C_5_–H, *J* = 7.8 Hz), 11.87 (1H, s, NH, D_2_O exchangeable); ^13^C NMR (CDCl_3_) *δ*_C_: 22.70, 25.13, 45.86, 112.53, 118.53, 119.89, 125.79, 127.49, 128.84, 142.9, 150.32, 161.15, 116.36, 135.92, 151.58, 169.17, 178.12, 207.06; MS (*m*/*z*): 402 M^+*∙*^ (found: C, 59.90; H, 4.32; N, 13.62%. C_20_H_17_F_3_N_4_O_2_ requires C, 59.70; H, 4.26; N, 13.92). 

*2-(2,7-Dimethyl-1,8-naphthyridin-4-yloxy)-N*′*-(4-methoxybenzylidene) Acetohydrazide (* ***4b*** *). *Off-white crystals; m.p. 256–258°C. FTIR: 1245 (–OCH_3_); 1605 (C=C); 1640 (C=N); 1679 (C=O amidic); 3100 (–NH). ^1^H NMR (600 MHz, CDCl_3_: DMSO-*d*_6_) *δ*_H_: 2.15, 2.46 (6H, 2s, 2CH_3_); 3.85 (3H, s, –OCH_3_); 5.77 (2H, br.s, –OCH_2_CO); 6.19 (1H, s, C_3_–H); 6.95, 7.65 (4H, 2*d*, *p*-disubstituted benzene *J* = 9.0 Hz); 7.20 (1H, *d*, C_6_–H, *J* = 7.8 Hz); 8.00 (1H, s, –N=CH); 8.46 (1H, *d*, C_5_–H, *J* = 7.8 Hz); 11.49 (1H, s, NH, D_2_O exchangeable); ^13^C NMR (CDCl_3_) *δ*_C_: 21.45, 25.13, 45.91, 55.46, 112.46, 114.41, 116.36, 118.52, 119.81, 125.76, 128.93, 135.84, 145, 150.39, 152.00, 161.77, 178.13, 207.04; MS (*m*/*z*): 364  M^+*∙*^ (found: C, 66.03; H, 5.33; N, 15.46. C_20_H_20_N_4_O_3_ requires C, 65.92; H, 5.53; N, 15.38).

*2-(2,7-Dimethyl-1,8-naphthyridin-4-yloxy)-N*′*-(furan-2-ylmethylene) Acetohydrazide (* ***4c*** *). *Off-white crystals; m.p. 217–219°C. FTIR: 1600 (C=C); 1631 (C=N); 1682 (C=O amidic); 3200 (–NH). ^1^H NMR (600 MHz, CDCl_3_: DMSO-*d*_6_) *δ*_H_: 2.17, 2.46 (6H, 2s, 2CH_3_), 5.78 (2H, br.s, –CH_2_), 6.22 (1H, s, C_3_–H), 7.18 (1H, *d*, C_6_–H, *J* = 7.8 Hz), 8.48 (1H, *d*, C_5_–H, *J* = 7.8 Hz); 6.53 (1H, dd, C_4_–H), 6.75 (1H, *d*, C_5_′–H), 7.56 (1H, *d*, C_3_′–H), 7.95 (1H, s, –N=CH–) and 11.53 (1H, s, N–H, D_2_O exchangeable); ^13^C NMR (CDCl_3_) *δ*_C_: 21.41, 25.12, 45.92, 112.13, 112.46, 113.89, 116.36, 118.50, 119.81, 134.22, 135, 144.97, 151.64, 161.77, 168.96, 178.00, 207.04; MS (*m*/*z*): 324  M^+*∙*^ (found: C, 63.11; H, 4.63; N, 17.03. C_17_H_16_N_4_O_3_ requires C, 62.95; H, 4.97; N, 17.27). 

*N*′*-(Benzo[d][1,3]dioxol-5-yl methylene)-2-(2,7-dimethyl-1,8-naphthyridin-4-yloxy) Acetohydrazide (* ***4d*** *). *Yellow crystals; m.p. 258-259°C. FTIR: 1247 (ether linkage); 1604 (C=C), 1630 (C=N), 1684 (C=O amidic), 3182 (NH); ^1^H NMR (600 MHz, CDCl_3_: DMSO-*d*_6_) *δ*_H_: 2.16, 2.48 (6H, 2s, 2CH_3_), 5.76 (2H, br.s, –OCH_2_CO), 6.02 (2H, dd, –OCH_2_O, *J* = 12.6 Hz), 6.85, 7.34, 7.58 (3H, trisubstituted benzene); 7.95 (1H, s, –CH=N–) and 11.43 (1H, s, –NH, D_2_O exchangeable). ^13^C NMR (CDCl_3_) *δ*_C_: 21.43, 25, 45.89, 101, 105.48, 106.62, 108.4, 125.23, 148.33, 150.32, 112.48, 116.37, 119.81, 123.87, 128.78, 144.31, 152, 161.22, 178.00, 207.04; MS (*m*/*z*): 378  M^+*∙*^ (found: C, 63.12; H, 5.43; N, 14.61. C_20_H_18_N_4_O_4_ requires C, 63.48; H, 4.79; N, 14.81).

*N*′*-(3,4-Dimethoxybenzylidene)-2-(2,7-dimethyl-1,8-naphthyridin-4-yloxy) Acetohydrazide (* ***4e*** *). *Yellow crystals; m.p. 269.5–270.8°C. FTIR: 1264 (ether linkage); 1600 (C=N), 1603 (C=C), 1682 (C=O amidic), 3120 (NH).^1^H NMR (600 MHz, CDCl_3_: DMSO-*d*_6_) *δ*_H_: 2.47, 2.56 (6H, 2s, 2CH_3_); 3.95, 3.98 (6H, 2s, 2–OCH_3_), 5.80 (2H, br.s, –CH_2_), from 6.30 to 10.10 (8H, m, 6 CH, N=CH, and NH). ^13^C NMR (CDCl_3_) *δ*_C_: 21.48, 25.15, 45.92, 48.00, 56.017, 112.45, 118.50, 119.83, 122.45, 124.00, 126.03, 127.24, 135.83, 145.13, 150.40, 151.49, 151.81, 161.79, 169.06 178.12, 207.06; MS (*m*/*z*): 394  M^+*∙*^ (found: C, 63.95; H, 5.43; N, 14.03. C_21_H_22_N_4_O_4_ requires C, 63.55; H, 5.62; N, 14.20).

*4.1.2.3. Synthesis of 2-(2,7-Dimethyl-1,8-naphthyridin-4-yloxy) Acetohydrazide—Aromatic Ketones Schiff's Bases (* ***5a***, ***5b***)

*4.1.2.3.1. Method A: Silent Reactions. *An equimolar mixture of (**3**) (0.004 moL) and the appropriate aromatic heterocyclic ketones, namely, a 2-acetylthiophene and 2-acetylfuran (0.004 moL) in 20 mL absolute ethanol, were stirred under reflux for 6 h. The reaction mixture was concentrated and cooled and the formed precipitate was recrystallized from ethanol/petroleum ether to give the title product **5a**, **5b**.

*4.1.2.2.2. Method B: Sonicated Reactions. *To a solution of 2-(2,7-dimethyl-1,8-naphthyridin-4-yloxy) acetohydrazide **3** (0.004 moL) in ethanol (20 mL) in a 100 mL Erlenmeyer flask, an appropriate aromatic heterocyclic ketone (0.004 moL) was added. The mixture was subjected to ultrasound irradiation for a suitable time (cf. Table 1) until the starting materials were no longer detectable by TLC. The reaction was kept at temperature 60–65°C which was attained by addition or removal of water in ultrasonic bath, (the temperature inside the reaction vessel was 60°C). The precipitate formed was filtered off and recrystallized from ethanol/pet. ether to afford the corresponding Schiff's bases **5a**, **5b**.

*2-(2,7-Dimethyl-1,8-naphthyridin-4-yloxy)-N*′*-(1-(thiophen-2-yl)ethylidene) Acetohydrazide (* ***5a*** *). *Yellow crystals; m.p. 234−236°C. FTIR: 1619 (C=C); 1680 (C=N amidic); (1694 C=O); 3228 (N−H); ^1^H NMR (600 MHz, CDCl_3_) *δ*_H_: 2.17, 2.42, 2.56 (9H, 3s, 3–CH_3_), 5.75 (2H, br.s, –CH_2_), 6.25 (1H, s, C_3_−H), 7.12 (1H, *d*, C_6_−H, *J* = 7.8 Hz), 8.55 (1H, *d*, C_5_−H, *J* = 7.8 Hz); three thiophene protons appear at 7.06 (1H, dd, C_4_′−H), 7.32 (1H, *d*, C_5_′−H), 7.37 (1H, *d*, C_3_′−H), 9.15 (1H, br.s, –NH, D_2_O exchangeable); ^13^C NMR (CDCl_3_) *δ*_C_: 13.06, 21.46, 25.15, 45.92, 112.42, 118.49, 119.80, 127.54, 128.35, 135.82, 142.75, 144.83, 150.40, 151.73, 161.81, 169, 178, 207.05; MS (*m*/*z*): 354  M^+*∙*^ (found: C,: C, 61.00; H, 5.12; N, 15.81. C_18_H_18_N_4_O_2_S requires C, 61.36; H, 5.33; N, 15.96). 

*2-(2,7-Dimethyl-1,8-naphthyridin-4-yloxy)-N*′*-(1-(furan-2-yl)ethylidene) Acetohydrazide (* ***5b*** *). *Yellow crystals, m.p 173–175°C. FTIR: 1600 (C=C); 1617 (C=N), 1671 (C=O amidic), 3216 (N–H); ^1^H NMR (600 MHz, CDCl_3_) *δ*_H_: 2.17, 2.46, 2.57 (9H, 3s, 3–CH_3_), 5.76 (2H, br.s, CH_2_), 6.27 (1H, s, C_3_–H), 7.15 (1H, *d*, C_6_–H, *J* = 7.8 Hz), 8.55 (1H, *d*, C_5_–H, *J* = 7.8 Hz), 7.05 (1H, dd, C_4_′–H), 7.33 (1H, *d*, C_5_′–H), 7.36 (1H, *d*, C_3_′–H), 9.00 (1H, br.s, –NH, D_2_O exchangeable); ^13^C NMR (CDCl_3_) *δ*_C_: 12.05, 21.45, 25.13, 46.08, 110.84, 111.91, 112.31, 118.45, 119.73, 135.79, 141.21, 144.13, 150.41, 151.81, 161.81, 169.70, 178.13, 207.09; MS (*m*/*z*): 338  M^+*∙*^ (found: C, 64.09; H, 5.66; N, 16.36. C_18_H_18_N_4_O_3_ requires C, 63.98; H, 5.36; N, 16.56).

*4.1.2.3. 2-(2,7-Dimethyl-1,8-naphthyridin-4-yloxy)-N*′*-(2-oxoindolin-3-ylidene) Acetohydrazide (* ***6***)

*4.1.2.3.1. Method A: Silent Reactions. *A mixture of isatin (0.01 moL) and acid hydrazide **3** (0.01 moL) in ethanol, acidified with 4 drops of glacial acetic acid, was refluxed for 1 h. The reaction mixture was then concentrated, cooled, and filtered. The filtered precipitate was washed thoroughly with ether and recrystallized from dilute ethanol to give **6 **as brown crystals.

*4.1.2.3.2. Method B: Sonicated Reactions. *A solution (0.01 moL) of isatin and acid hydrazide **3** (0.01 moL) in ethanol, acidified with 4 drops of glacial acetic acid in a 100 mL Erlenmeyer flask, was subjected to ultrasound irradiation for 10 minutes. The reaction was kept at a temperature of 60–65°C which was attained by addition or removal of water in the ultrasonic bath (the temperature inside the reaction vessel was 60°C). The filtered precipitate was washed thoroughly with ether and recrystallized from dilute ethanol to produce the corresponding Schiff's bases **6 **as brown crystals; m.p. 298–301°C. FTIR; 3154, 3214 (NH, OH); 1685 (C=O amidic); ^1^H NMR (600 MHz, DMSO-*d*_6_) *δ*_H_: 2.37, 2.42 (6H, 2s, 2CH_3_), 5.80 (2H, br.s, –OCH_2_CO), 6.15–8.35 (7H, m., aromatic CH), 10.88 (1H, br.s, –NH of isatin), 11.3 (1H, br.s, NH amidic) and 11.62, 12.72 (2H, 2br.s, enolic OH); ^13^C NMR (DMSO-*d*_6_) *δ*_C_: 20.80, 24.80, 45.36, 111.26, 115.17, 117.92, 119.77, 122.7, 126.30, 131.96, 135.23, 142.70, 143.94, 149.96, 153.00, 161.45, 164.45, 169.78, 176.25, 207.00; MS (*m*/*z*): 375  M^+*∙*^ (found: C, 64.12; H, 4.23; N, 18.32. C_20_H_17_N_5_O_3_ requires C, 63.99; H, 4.56; N, 18.66).

*4.1.2.4. Synthesis of N-Acetyl-2-(2,7-dimethyl-1,8-naphthyridin-4-yloxy)-N-(4-oxo-2-aryl)-thiazolidin-3-yl) Acetamide (* ***7a-d*** *)*

*4.1.2.4.1. Method A: Silent Reaction. *A mixture of Schiff's bases **4a**–**d **(0.001 moL) and (0.4 mL, 0.001 moL) thioglycolic acid in 8 mL glacial acetic acid was refluxed for 36–48 h (until disappearance of the starting materials as examined by TLC) (cf. Table 2). The reaction mixture was cooled at r.t., neutralized with ammonia and the precipitate formed was collected, filtered, and crystallized from ethanol.

*4.1.2.4.2. Sonicated Reaction. *A solution of Schiff's bases **4a**–**d **(0.001 moL) in 8 mL glacial acetic acid and (0.4 mL, 0.001 moL) thioglycolic acid in a 50 mL Erlenmeyer flask was subjected to ultrasound irradiation for a suitable time (cf. Table 2) until the starting material was no longer detectable by TLC. The reaction was kept at 70–75°C which was attained by addition or removal of water in the ultrasonic bath (the temperature inside the reaction vessel was 70°C). The reaction mixture was cooled to r.t. and neutralized with ammonia and the precipitate formed was collected, filtered, and crystallized from ethanol.

*4.1.2.4.3. Catalyzed Sonicated Reaction. *To a solution of Schiff's bases **4a**–**d **(0.001 moL) in 8 mL glacial acetic acid and thioglycolic acid (0.4 mL and 0.001 moL) in a 50 mL Erlenmeyer flask, a molecular sieve (1 g, 4 Å) was added. The mixture was subjected to ultrasound irradiation for a suitable time (cf. Table 2) until the starting material was no longer detectable by TLC. The reactants were kept at 70–75°C which was attained by addition or removal of water in the ultrasonic bath (the temperature inside the reaction vessel was 70°C). The reaction mixture was decanted, cooled, and neutralized with ammonia. The formed precipitate was collected, filtered, and crystallized from ethanol.

The synthesized compounds (**7a**–**d**) with their physical data are listed below.

*N-Acetyl-2-(2,7-dimethyl-1,8-naphthyridin-4-yloxy)-N-(4-oxo-2-(4-(trifluoromethyl)phenyl)thiazolidin-3-yl) Acetamide (* ***7a*** *).* Yellow crystals, m.p. 306–308°C. FTIR: 1608 (C=C), 1620 (C=N);, 1678 (C=O amidic), 3366 (–OH enolic); ^1^H NMR (600 MHz, DMSO-*d*_6_) *δ*_H_: 2.07 (3H, s, –COCH_3_); 2.37, 2.41 (6H, 2S, 2 CH_3_); 3.71, 3.90 (2H, 2*d*, –CH_2_– of thiazolidine ring *J* = 16.2 Hz), 5.90 (2H, s, –CH_2_), 6.07 (1H, s, C_3_–H), 7.24 (1H, *d*, C_6_–H, *J* = 7.8 Hz), 7.66, 7.75 (4H, 2*d*, for *p*-disubstituted benzene *J* = 8.4 Hz), 8.29 (1H, s, C_2_–H of thiazolidinone), 8.39 (1H, *d*, C_5_–H, *J* = 7.8 Hz). ^13^C NMR (DMSO-*d*_6_) *δ*_C_: 20.73, 24.39, 24.79, 30.70, 45.08, 60.72, 111.18, 117.74, 119.65, 123.17, 125.60, 127.60, 128.38, 129.23, 135.07, 149.80, 152.78, 161.39, 166.82, 176.14, 193.42, and 207.00; MS (*m*/*z*): 518  M^+*∙*^ (found: C, 55.49; H, 4.28; N, 10.21. C_24_H_21_F_3_N_4_O_4_S requires C, 55.59; H, 3.96; N, 10.53).

*N-Acetyl-2-(2,7-dimethyl-1,8-naphthyridin-4-yloxy)-N-(2-(4-methoxyphenyl)-4-oxothiazolidin-3-yl) Acetamide (* ***7b*** *). *Yellow crystals, m.p 293–295°C. FTIR: 1245 (ether linkage); 1680 (C=O amidic); 3361 (OH enolic); ^1^H NMR (600 MHz, DMSO-*d*_6_) *δ*_H_: 2.06, 2.37, 2.54 (9H, 3s, 3CH_3_); 3.60, 3.75 (2H, dd, CH_2_ of thiazolidinone ring, *J* = 15.6 Hz); 3.84 (3H, s, –OCH_3_); 5.95 (2H, br.s, –CH_2_); 6.08–8.39 (8H, m., for *p*-disubstituted benzene, naphthyridine, and C_2_–H of thiazolidinone). ^13^C NMR (DMSO-*d*_6_) *δ*_C_: 20.49, 24.39, 28.87, 30.74, 45.16, 46.10, 55.77, 111.20, 117.75, 119.73, 120.74, 122.20, 126.43, 129.99, 135.12, 139.98, 149.87, 152.91, 161.50, 168.73, 176.21, and 206.64; MS (*m*/*z*): 480  M^+*∙*^ (found: C, 60.29; H, 4.51; N, 11.39. C_24_H_24_N_4_O_5_S requires C, 59.99; H, 5.03; N, 11.66).

*N-Acetyl-2-(2,7-dimethyl-1,8-naphthyridin-4-yloxy)-N-(2-(furan-2-yl)-4-oxothiazolidin-3-yl) Acetamide (* ***7c*** *). *Off-white crystals, m.p. 241–243°C. FTIR; 1631 (C=N), 1681 (C=O amidic), 3460 (–OH enolic). ^1^H NMR (600 MHz, DMSO-*d*_6_) *δ*_H_: 2.07 (3H, s, COCH_3_); 2.36, 2.52 (6H, 2s, 2CH_3_); 3.66, 3.80 (2H, 2*d*, CH_2_ of thiazolidinone ring, *J *= 16.2 Hz), 5.86 (2H, br.s, OCH_2_), 6.12 (1H, s, C_3_–H), 6.46 (1H, dd, C_4_′–H, *J*_4′,3′_= 3 Hz, *J*_4′,5′_ = 1.8 Hz), 6.54 (1H,* d*, C_3_′–H, *J*_3′,4′_ = 3 Hz), 7.27 (1H, *d*, C_6_–H, *J* = 7.8 Hz), 7.73 (1H, *d*, C_5_′–H, *J*_5′,4′_= 1.8 Hz), 7.96 (1H, s, CH of thiazolidinone ring), 8.31 (1H, *d*, C_5_–H, *J* = 7.8 Hz). ^13^C NMR (DMSO-*d*_6_) *δ*_C_: 20.50, 24.60, 28.60, 30.69, 45.02, 54.58, 110.22, 110.61, 111.21, 117.77, 119.79, 135.09, 144.25, 148.89, 149.87, 150.01, 152.78, 161.32, 168.17, 176.15, and 206.53; MS (*m*/*z*): 440  M^+*∙*^ (found: C, 57.56; H, 4.28; N, 12.42. C_21_H_20_N_4_O_5_S requires C, 57.26; H, 4.58; N, 12.72).

*N-Acetyl-N-(2-(benzo[d][1,3]dioxol-5-yl)-4-oxothiazolidin-3-yl)-2-(2,7-dimethyl-1,8-naphthyridin-4-yloxy) Acetamide (* ***7d*** *). *Yellow crystals, m.p. 296–298°C. FTIR: 1603 (C=N), 1680 (C=O amidic), 3480 (–OH). ^1^H NMR (600 MHz, DMSO-*d*_6_) *δ*_H_: 2.07 (3H, s, –CH_3_CO), 2.48, 2.55 (6H, 2s, 2CH_3_), 3.41, 3.47 (2H, 2*d*, CH_2_ of thiazolidine ring), 5.70 (2H, br.s, OCH_2_), 6.08 (2H, s, OCH_2_O), 6.12 (1H, s, C_3_–H), 6.96, 7.16, 7.39 (1H, m, 3CH of benzene), 6.54 (1H, *d*, C_3_′–H, *J*_3′,4′_ = 3 Hz), 7.28 (1H, *d*, C_6_–H, *J* = 7.8 Hz), 7.97 (1H, s, CH), 8.36 (1H, *d*, C_5_–H, *J* = 7.8 Hz); ^13^C NMR (DMSO-*d*_6_) *δ*_C_: 20.68, 24.76, 30.68, 40.02, 41.85, 45.63, 101.54, 105.13, 108.43, 111.06, 117.86, 119.56, 113.24, 128.41, 135.11, 143.88, 147.92, 150.12, 153.03, 161.30, 168.69, 171.99, 176.12, and 206.50; MS (*m*/*z*): 494  M^+*∙*^ (found: C, 58.56; H, 4.28; N, 11.42. C_24_H_22_N_4_O_6_S requires C, 58.29; H, 4.45; N, 11.33).

*4.1.2.5. 4-Bromo-2-(2-((2,7-dimethyl-1,8-naphthyridin-4-yl)oxy)acetyl)-5-(4-methoxy phenyl)pyrazolidin-3-one (* ***8***)

*4.1.2.5.1. Method A: Silent Reaction. *A mixture of Schiff's base **4b** (0.36 gm, 0.001 moL) and (0.09 mL 0.001 moL) bromoacetyl bromide in 10 mL absolute ethanol and MgO (0.5 gm) was refluxed for 72 h (until the disappearance of starting materials as examined by TLC). The reaction mixture was cooled; the precipitate formed was filtered off, washed with ethanol, and recrystallized from ethanol/DMF to produce the title compound in a yield of 45%.

*4.1.2.5.2. Sonicated Reaction. *A solution of 2-(2,7-dimethyl-1,8-naphthyridin-4-yloxy)-*N*′-(4-methoxybenzylidene) acetohydrazide **4b** (0.36 gm, 0.001 moL), (0.09 mL, 0.001 moL) bromoacetyl bromide in 10 mL absolute ethanol, and MgO (0.5 gm) in a 50 mL Erlenmeyer flask was subjected to ultrasound irradiation for 10 min until the starting material was no longer detectable by TLC. The reaction was kept at 70–75°C which was attained by addition or removal of water in the ultrasonic bath (the temperature inside the reaction vessel was 70°C). The reaction mixture was cooled at r.t.; the precipitate formed was filtered off, washed with ethanol, and recrystallized from ethanol/DMF to produce compound **8** in a yield of 96%. Characteristics: m.p. 237–239°C. FTIR: 3338 (–NH), 1676 (C=O amidic), 1605 cm^−1^ (C=N); ^1^H NMR (600 MHz, DMSO-*d*_6_) *δ*_H_: 2.39, 2.61 (6H, 2s, 2CH_3_), 2.99, 3.32 (2H, 2*d*, 2CH of pyrazolidinone), 3.70 (3H, s, OCH_3_), 5.80 (2H, s, –OCH_2_), 6.20 −8.49 (8H, m, ArH^,^s,NH); ^13^C NMR (EtOD) *δ*_C_: 18.43, 21.43, 25.11, 30.94, 45.83, 54.09, 112.26, 118.43, 119.77, 123.80, 128.36, 129.17 135.86, 142.86, 150.29, 155.93, 161.59, 166.27, 178.03, 207.06; MS (*m*/*z*): 485M^+*∙*^, 487M^+*∙*^ + 2 (found: C, 54.81; H, 4.02; N, 11.91. C_22_H_21_Br N_4_O_4_ requires C, 54.44; H, 4.36; N, 11.54).

### 4.2. Cytotoxicity

#### 4.2.1. Measurement of Potential Cytotoxicity by SRB Assay

The selected 1,8-naphthyridine derivatives compounds (**3**,** 4a**,** 4b**,** 4c**,** 4d**,** 7a**,** 7b**,** 7c**, and** 7d**) were subjected to a screening system for evaluation of their antitumor activity against the liver HepG2 cancer cell line in comparison to the known anticancer drug DOX. The potential cytotoxicity of the selected 1,8-naphthyridine derivatives was tested using the method of Skehan et al. [31] as follows: cells were plated in a 96-multiwell plate (10^4^ cells/well) for 24 h before treatment with the compounds to allow attachment of the cells to the wall of the plate. Different concentrations of the compound under test (5, 12.5, 25, and 50 *μ*g/mL) were added to the cell monolayer. Triplicate wells were prepared for each individual dose. Monolayer cells were incubated with the compounds for 48 h at 37°C and in an atmosphere of 5% CO_2_. Cultures were fixed with trichloroacetic acid and stained for 30 min with 0.4% (wt/vol.) sulforhodamine B (SRB) dissolved in 1% acetic acid. Unbound dye was removed by four washes with 1% acetic acid, and protein-bound dye was extracted with 10 *μ*M unbuffered tris base [tris(hydroxymethyl)aminomethane] for determination of optical density in a computer-interfaced, 96-well microtiter plate reader. The SRB assay results were linear with the number of cells and with values for cellular protein measured by both the Lowry and Bradford assays at densities ranging from sparse subconfluence to multilayered supraconfluence. The signal-to-noise ratio at 564 nm was approximately 1.5 with 1,000 cells per well. The relation between the surviving fraction and drug concentration is plotted to get the survival curve of both cancer cell lines after treatment with the specified compound.

## Figures and Tables

**Table 1:** Synthesis of Schiff's bases derivatives **4a**–**e**, **5a**, **5b**, and **6 **both under ultrasonic irradiation and using the conventional method.

Compound	Ultrasonic irradiation		Conventional	
	Time (min.)	Yield %	Time (h)	Yield %
**4a**	15	98	1	97
**4b**	30	93	2	91
**4c**	30	93	2	91
**4d**	20	96	2	94
**4e**	30	93	3	92
**5a**	40	95	6	93
**5b**	40	91	8	88
**6**	10	98	1	96

**Table 2:** Synthesis of 4-oxothiazolidine derivatives (**7a**–**d**) under catalyzed ultrasonic irradiation, uncatalyzed ultrasonic irradiation, and using the conventional method.

Compound **7**	Catalyzed ultrasonic irradiation		Uncatalyzed ultrasonic irradiation		Conventional	
	Time (min)	Yield %	Time (min)	Yield %	Time (h)	Yield %
**a**	40	90	100	43	36	40
**b**	60	89	100	51	44	43
**c**	40	93	85	47	48	44
**d**	65	92	90	56	39	49

**Table 3:** Cytotoxic activity of the newly synthesized selected derivatives on the liver HepG2 cancer cell line in comparison to the traditional anticancer drug DOX.

Compounds	IC_50_ (*μ*M)
**3**	0.086
**4a**	0.047
**4b**	0.057
**4c**	0.094
**4d**	0.053
**7a**	0.052
**7b**	0.041
**7c**	0.053
**7d**	0.049
DOX	0.040

## References

[1] Rees CW, Katrizky AR, Scriven EFV (1996). *Comprehensive Heterocyclic Chemistry II*.

[2] Massari S, Daelemans D, Barreca ML (2010). A 1,8-naphthyridone derivative targets the HIV-1 Tat-mediated transcription and potently inhibits the HIV-1 replication. *Journal of Medicinal Chemistry*.

[3] Fadda AA, El-Defrawy AM, El-Habiby SA (2012). Synthesis, cytotoxicity evaluation, DFT molecular modeling studies and quantitative structure activity relationship of novel 1, 8-naphthyridines. *American Journal of Organic Chemistry*.

[4] Roma G, Grossi G, di Braccio M (2008). 1,8-naphthyridines VII. New substituted 5-amino[1,2,4]triazolo[4,3-*a*][1,8]naphthyridine-6-carboxamides and their isosteric analogues, exhibiting notable anti-inflammatory and/or analgesic activities, but no acute gastrolesivity. *European Journal of Medicinal Chemistry*.

[5] Olepu S, Suryadevara PK, Rivas K (2008). 2-Oxo-tetrahydro-1,8-naphthyridines as selective inhibitors of malarial protein farnesyltransferase and as anti-malarials. *Bioorganic & Medicinal Chemistry Letters*.

[6] Laxminarayana E, Karunakar T, Shankar SS, Chary MT (2012). A study on antibacterial activity of substituted 1,8-naphthyridines containing carbaldehydes, methyleden hydrazines, thiadiazolamines and triazolethiols. *Journal of Advances in Drug Research*.

[7] Quintela JM, Peinador C, González L (2003). Piperazine N-substituted naphthyridines, pyridothienopyrimidines and pyridothienotriazines: new antiprotozoals active against *Philasterides dicentrarchi*. *European Journal of Medicinal Chemistry*.

[8] Aboul-Fadl T, Bin-Jubair FAS, Aboul-Wafa O (2010). Schiff bases of indoline-2,3-dione (isatin) derivatives and nalidixic acid carbohydrazide, synthesis, antitubercular activity and pharmacophoric model building. *European Journal of Medicinal Chemistry*.

[9] Ferrarini PL, Mori C, Badawneh M (2000). Synthesis and antiplatelet activity of some 3-phenyl-1,8-naphthyridine derivatives. *II Farmaco*.

[10] Kren V, Martinkova L (2001). The role of glycosidic residue in biological activity. *Current Medicinal Chemistry*.

[11] Křen V, Řezanka T (2008). Sweet antibiotics—the role of glycosidic residues in antibiotic and antitumor activity and their randomization. *FEMS Microbiology Reviews*.

[12] Tsuzuki Y, Tomita K, Sato Y, Kashimoto S, Chiba K (2004). Synthesis and structure-activity relationships of 3-substituted 1,4-dihydro-4-oxo-1-(2-thiazolyl)-1,8-naphthyridines as novel antitumor agents. *Bioorganic & Medicinal Chemistry Letters*.

[13] Burden DA, Osheroff N (1998). Mechanism of action of eukaryotic topoisomerase II and drugs targeted to the enzyme. *Biochimica et Biophysica Acta*.

[14] Eweas AF, Khalifa NM, Ismail NS, Al-Omar IMA, Soliman AM (2014). Synthesis, molecular docking of novel 1,8-naphthyridine derivatives and their cytotoxic activity against HepG2 cell lines. *Medicinal Chemistry Research*.

[15] Cravotto G, Cintas P (2006). Power ultrasound in organic synthesis: moving cavitational chemistry from academia to innovative and large-scale applications. *Chemical Society Reviews*.

[16] Pizzuti L, Martins PL, Ribeiro BA (2010). Efficient sonochemical synthesis of novel 3,5-diaryl-4,5-dihydro-1H-pyrazole-1-carboximidamides. *Ultrasonics Sonochemistry*.

[17] Zhengyan F, Huawu S (2011). An efficient synthesis of 3-substituted indole derivates under ultrasound irradiation. *Ultrasonics Sonochemistry*.

[18] Liu Q, Ai H, Li Z (2011). Potassium sorbate as an efficient and green catalyst for Knoevenagel condensation. *Ultrasonics Sonochemistry*.

[19] Jadidi K, Gharemanzadeh R, Mehrdad M, Darabi HR, Khavasi HR, Asgari D (2008). A facile synthesis of novel pyrrolizidines under classical and ultrasonic conditions. *Ultrasonics Sonochemistry*.

[20] Shaaban MR, Saleh TS, Mayhoub AS, Farag AM (2011). Single step synthesis of new fused pyrimidine derivatives and their evaluation as potent Aurora-A kinase inhibitors. *European Journal of Medicinal Chemistry*.

[21] Ahmed NS, Saleh TS, El-Mossalamy EH (2013). An efficiently sonochemical synthesis of novel pyrazoles, bipyrazoles and pyrazol-3-ylpyrazolo[3,4-d]pyrimidines incorporating 1H-benzoimidazole. *Current Organic Chemistry*.

[22] Christopher JC, Deady LW, James AR, Tzimos V (1982). The synthesis of macrocyclic polyether-diesters incorporating 1,10-phenanthrolino and 1,8-naphthyridino subunits. *Journal of Heterocyclic Chemistry*.

[23] Holla BS, Kalluraya B, Nath SC (1988). Synthesis, characterization, spectral and antifungal properties of some 5-Substituted-1,3,4-oxadiazole-2-thiones and their mannich bases. *Journal für Praktische Chemie*.

[24] Sridhar S, Saravanan M, Ramesh A (2001). Synthesis and antibacterial screening of hydrazones, Schiff and Mannich bases of isatin derivatives. *European Journal of Medicinal Chemistry*.

[25] Robin M, Bovey F, Basch H, Zabicky J (1970). Molecular and electronic structure of the amide group. *The Chemistry of Amides*.

[26] Wu TY, Guo N, Teh CY, Hay JXW (2013). *Advances in Ultrasound Technology for Environmental Remediation*.

[27] Cabello N, Cintas P, Luche J-L (2003). Sonochemical effects in the additions of furan to masked ortho-benzoquinones. *Ultrasonics Sonochemistry*.

[28] Kyu-Sung J, Cho YL (1997). Highly strong complexation of carboxylates with 1-alkylpyridinium receptors in polar solvents. *Tetrahedron Letters*.

[29] Loupy A, Luche JL, Luche JL (1998). Sonochemistry in biphasic systems. *Synthetic Organic Sonochemistry*.

[30] Suslick KS (1990). Sonochemistry. *Science*.

[31] Skehan P, Storeng R, Scudiero D (1990). New colorimetric cytotoxicity assay for anticancer-drug screening. *Journal of the National Cancer Institute*.

